# Metagenomic analysis of formalin-fixed paraffin-embedded tumor and normal mucosa reveals differences in the microbiome of colorectal cancer patients

**DOI:** 10.1038/s41598-020-79874-y

**Published:** 2021-01-11

**Authors:** Gabriela Debesa-Tur, Vicente Pérez-Brocal, Susana Ruiz-Ruiz, Adela Castillejo, Amparo Latorre, José Luis Soto, Andrés Moya

**Affiliations:** 1 Fundación para el Fomento de la Investigación Sanitaria y Biomédica de la Comunitat Valenciana (FISABIO), Área de Genómica y Salud, Valencia, Spain; 2 Consorcio de Investigación Biomédica en Red de Epidemiología y Salud Pública (CIBERESP), Madrid, Spain; 3 Unidad de Genética Molecular, Hospital General Universitario de Elche, Alicante, Spain; 4 Departamento de Salud Elche, Fundación para el Fomento de la Investigación Sanitaria y Biomédica de la Comunitat Valenciana (FISABIO), Elche, Spain; 5 Instituto de Biología Integrativa de Sistemas (I2Sysbio), Universitat de València and Consejo Superior de Investigaciones Científicas (CSIC), Paterna, Valencia, Spain

**Keywords:** Colorectal cancer, Microbial genetics, Sequencing, Microbial communities, Clinical microbiology, Metagenomics, Microbiome, Cancer, Microbiology

## Abstract

An increased risk of developing colorectal cancer (CRC) and other types of tumor is associated to Lynch syndrome (LS), an inherited condition caused by germline mutations in mismatch repair genes. We selected a cohort of LS patients that had developed CRC and had undergone surgical resection. Formalin-fixed paraffin embedded (FFPE) tissue blocks from matched colorectal and normal mucosa were used for genomic DNA extraction with a commercial kit and sequenced by high-throughput sequencing. A metagenomic approach enabled the taxonomic and functional identification of the microbial community and associated genes detected in the specimens. Slightly lower taxonomic diversity was observed in the tumor compared to the non-tumor tissue. Furthermore, the most remarkable differences between tumors and healthy tissue was the significant increase in the genus *Fusobacterium* in the former, in particular the species *F. nucleatum*, as well as *Camplylobacter* or *Bacteroides fragilis*, in accordance with previous studies of CRC. However, unlike prior studies, the present work is not based on directed detection by qPCR but instead uses a metagenomic approach to retrieve the whole bacterial community, and addresses the additional difficulty of using long-term stored FFPE samples.

## Introduction

Colorectal cancer (CRC) is the third most common cancer worldwide, with an incidence over 1.8 million new cases in 2018, and second in mortality with over 860,000 deaths in 2018^1^. Moreover, according to the Global Cancer Observatory (https://gco.iarc.fr/today/data/factsheets/populations/724-spain-fact-sheets.pdf), in Spain, CRC occupied the first position in terms of incidence in 2018, with an estimated 36,689 new cases (13.5% of all cases), and ranking second in mortality, with 16,568 deaths (14.5% of all deaths by cancer). This type of cancer is more frequent in men (21.1 cases per 100,000) than in women (12.4 cases per 100,000), with both sexes suffering a steady increase in the last decades^2^. Although genetic predisposition syndromes for CRC, which include syndromes such as familial adenomatous polyposis (FAP), Peutz–Jeghers syndrome (PJS), juvenile polyposis syndrome and Lynch syndrome (LS), and other hereditary non-polyposis colorectal cancer conditions^3,4^ only account for a minority of cases, in families with members diagnosed with a familial CRC syndrome, the risk of CRC is much higher than in the general population. In particular, LS-associated CRC is a genetic subtype of cancer ultimately caused by germline inactivating mutations in the DNA mismatch repair genes (MMR), involved in recognition, removal and correction of mismatched DNA base pairs, rendering deficient mismatch repair and allowing mutations to accumulate^5^. MMR genes involved include MLH1, MSH2, MSH6, and PMS2. Approximately 2–5% of all cases of CRC are thought to be due to LS^6,7^), often with early onset (average age of 44–61 years). Lynch sydrome also includes other extracolonic manifestations, such as elevated risk of other cancers such as endometrial, ovarian, stomach, renal, ureter, small intestine, biliary, sebaceous gland tumors and glioblastoma^8^.

The role played by the human gut microbiota in health and in pathological conditions has been extensively studied, including its links with CRC (for an extensive review see^9^, with research dating back as early as the 1960s). Comparative 16S ribosomal RNA sequencing or metagenomic approaches on fecal or mucosal samples have been used in the last decade to investigate the role of the gut microbiota in CRC, showing differences between the microbiota of the former and healthy individuals used as controls (e.g.^10–15^.), with substantial changes in abundance of specific bacteria. From these and other studies, evidence is growing that the close interaction of microorganisms with host intestinal cells can affect the immunity and metabolome in the gut, and in the case of CRC the gut microbiota may be involved in CRC formation, progression and its response to treatment. Differences have also been observed between subtypes of CRC linked to deficient mismatch repair, including those associated with LS, and other subtypes of CRC with proficient mismatch repair genes^5^. However, most of the studies using mucosal tissue have been based on freshly frozen resected samples, which have advantages such as the relatively low level of handling and therefore fewer challenges associated with contamination or storage-derived issues, but also have the downside of relying on the immediacy and availability of specimens, which may be limiting for retrospective analyses, as well as requiring infrastructures for long-term sample storage. To overcome these drawbacks, having access to preserved tissue from collections deposited in biobanks allows the exploitation of samples from a wider range of locations and times, requiring relatively little effort to gather sufficient samples for epidemiologic studies, with the additional advantage of enabling the analysis of relatively old samples. In this regard, historical archives of matched formalin-fixed and paraffin embedded (FFPE) tumor and normal tissues from Hospital Pathology departments, available for research through Biobank networks, are an essential resource, particularly in rare pathological conditions such as LS. The challenge posed by this case resides in developing a methodology enabling us to extract information on the microbiota from samples predictably carrying considerable DNA alteration and degradation linked to fixation and preservation techniques.

In this study, we have carried out a comparative study of the microbiota from two FFPE tissue types (a distal macroscopically non-affected tissue and tumor tissue) from a cohort of patients from the Comunidad Valenciana (Spain), with previous genetic diagnosis of LS, and affected by colorectal cancer. Our aim is to assess the suitability of this type of sample for the metagenomic analysis of the associated microbiota in order to identify potential microbial biomarkers (both taxa and genes). This being the case, we further aimed to validate the comparability of our results with those of previous studies using fresh mucosal samples, thus allowing a greater range of biological samples to be made available for meta-omic studies of the microbiota.

## Results

### Cohort description and sequencing outcome

A total of 98 samples from 49 LS patients surgically treated between 1996 and 2017 were collected and sequenced. After processing, sequencing was successful for 47 out of the 49 pairs (i.e. sequences from both tumor and non-affected tissue were obtained) but failed in two cases for one sample of the pair (930937NT and 930946NT), thus obtaining 96 successful samples. Table 1 lists the patients with sample identification, tissue type (tumor or normal mucosa), mutated gene, patient gender and age, tumor location, grade of differentiation, TNM classification and stage is shown in. Patients in the study included 21 females (42.86%) and 28 males (57.14%), with an average age of 53.63 ± 12.52 years, ranging from 33 to 87 years. Regarding mutated genes, 19 individuals (38.78%) carried a mutation in MSH2; 16 (32.65%) in MLH1; 9 (18.37%) in MSH6; 4 (8.16%) in PMS2, and one individual (2.04%) carried a large deletion affecting genes MSH2 and MSH6. Information about tumor location was not provided for seven patients (14.29%), whereas for the remaining 42, the majority of biopsies were taken from the ascending colon (n = 24 representing 48.98%), compared to the remaining locations: transverse (n = 4 representing 8.16%), descending (n = 3 representing 6.12%), and sigmoid colon (n = 5 representing 10.20%), and rectum (n = 6 representing 12.24%). Due to the low number of samples in some groups, for statistical purposes, we grouped the ascending and transverse colon as a right-sided colon (RSC), and the descending and sigmoid colon as the left-sided colon (LSC). Therefore, the RSC group consisted of 28 samples, the LSC of 8 samples, and the 6 rectum samples as the third location. Regarding the type of tumor, there was a single adenoma (2.04%), 43 carcinomas (87.76%), plus 5 unknown cases (10.20%). All tumors with available information were reported as primary. According to the grade of differentiation, 15 (30.61%) were well differentiated, 17 (34.69%) moderately differentiated, and 8 (16.33%) poorly differentiated, with the remaining samples undetermined. Information on the stage was unavailable or unknown for 14 samples whereas 25 cases were classified as early stage (11 at stage I and 14 stage II), and the remaining 10 were assigned as advanced stage (all of them at stage III without any at stage IV).

**Table 1:** List of the patients with sample identification, year of collection, mutated gene, patient gender, age, tumor location, grade of differentiation, TNM classification and stage. Question mark (?) indicates data unknown or not provided.

Patient ID	Year	Mutated Gene	Gender	Age	Location	Grade of differentiation	TNM classification	Stage
930900	2007	MSH2	F	69	Rectum	Undetermined	?	?
930902	2015	MSH2	M	40	Ascending colon	Undetermined	T3N1	IIIB
930903	2014	MSH2	F	68	Ascending colon	Well differentiated	T2N0MX	I
930904	2015	MLH1	F	65	Ascending colon	Moderately differentiated	T1N0	I
930905	1996	MSH6	F	51	?	Well differentiated	?	?
930906	2008	MLH1	M	44	Rectum	Undetermined	T3N0	IIA
930907	2011	MSH2	M	70	Sigmoid colon	Well differentiated	T3N0	IIA
930908	2013	PMS2	M	57	Ascending colon	Moderately differentiated	T2N0	I
930909	2007	MLH1	M	42	Ascending colon	Poorly differentiated	T3N0	IIA
930910	2007	MLH1	F	48	?	Undetermined	?	?
930911	2006	MSH6	M	62	Ascending colon	Moderately differentiated	T3N0	IIA
930912	2013	PMS2	F	40	Ascending colon	Poorly differentiated	T3N2	IIIB
930913	2007	MSH2	M	47	?	Undetermined	?	?
930914	2011	MSH2	F	43	Ascending colon	Moderately differentiated	T2N1	IIIA
930915	2014	MSH6	F	80	Descending colon	Moderately differentiated	T3N1	IIIB
930916	2015	MLH1	F	41	Ascending colon	Well differentiated	T2N0	I
930917	2017	MSH2	F	77	Transverse colon	Poorly differentiated	T4N0MX	IIB
930918	2007	MLH1	F	58	Ascending colon	Moderately differentiated	?	?
930919	2007	MSH6	M	56	Ascending colon	Poorly differentiated	?	?
930920	2009	MSH2	M	47	Ascending colon	Well differentiated	T3N0M0	IIA
930921	2008	MLH1	F	74	Sigmoid colon	Moderately differentiated	T2N0M0	I
930922	2008	MLH1	M	61	Rectum	Moderately differentiated	T2N0	I
930923	2016	MSH2_MSH6	F	41	Ascending colon	Moderately differentiated	T1N0MX	I
930924	2000	MLH1	F	47	Ascending colon	Moderately differentiated	T2N0	I
930925	2007	MSH2	M	50	?	?	?	?
930926	2002	MLH1	M	61	?	Moderately differentiated	endoscopy	?
930927	2006	MSH6	M	43	Descending colon	Moderately differentiated	T3N1	IIIB
930928	2016	MSH2	M	54	Sigmoid colon	Moderately differentiated	T4N0	IIB
930929	2017	MSH2	M	55	Transverse colon	Well differentiated	T3N1	IIIB
930930	2013	MLH1	M	39	Ascending colon	Moderately differentiated	T3N0	IIA
930931	2017	MSH2	F	54	Ascending colon	Well differentiated	T3N0	IIA
930932	2007	MLH1	M	42	Ascending colon	Moderately differentiated	T3N0	IIA
930933	2013	MLH1	F	44	Ascending colon	Poorly differentiated	T3N0	IIA
930934	2017	MLH1	M	50	Transverse colon	Moderately differentiated	T3N0	IIA
930935	2010	MLH1	M	33	Transverse colon	Poorly differentiated	T4N2	IIIC
930936	2017	MSH2	F	49	Ascending colon	Undetermined	?	?
930937	2004	MSH6	M	69	Rectum	Poorly differentiated	T2N0M0	I
930938	2008	MSH6	M	46	Ascending colon	Well differentiated	T2N0M0	I
930939	2004	PMS2	F	62	Ascending colon	Well differentiated	T3N0M0	IIA
930940	2009	MSH6	M	66	Descending colon	Poorly differentiated	T2N0M0	I
930941	2009	MSH2	M	52	Sigmoid colon	Well differentiated	T4N2M0	IIIC
930942	2012	MSH2	M	59	?	?	?	?
930943	2016	MSH2	M	34	?	?	?	?
930944	2012	MSH6	F	62	Rectum	Well differentiated	T3N1	IIIB
930945	2015	MSH2	F	61	Ascending colon	Well differentiated	T3N2	III3B
930946	2016	MLH1	M	41	Ascending colon	Moderately differentiated	T3?	?
930947	2015	MSH2	F	87	Rectum	Well differentiated	T1?	?
930948	2013	MSH2	M	48	Ascending colon	Well differentiated	T3N0	IIA
930949	2009	PMS2	M	39	Sigmoid colon	Well differentiated	T3?	?

Despite obtaining good depth of sequencing in most samples, with 360,313,206 reads and an average of 3,714,569 ± 2,892,890 reads per sample, after filtering human-origin reads, the sequence reads of microbial origin decreased hugely, as expected, to 329,057 (or 0.091%) reads taxonomically assigned, with an average of 3427.68 ± 9,754.61 reads per sample, ranging from 17 to 73,995. On the other hand, 166,586 (or 0.046%) reads were functionally assigned, with an average of 1717.38 ± 3733.13 reads per sample, ranging from 55 to 21,880. Supplementary Table S1 shows the number of sequence reads per sample at different stages of the computational analysis.

Due to the considerable variability in the number of microbial sequence reads among the 96 samples, and in order to obtain comparable results, only two subsets of equal number of reads (200 and 400) were used from those samples containing at least those figures for subsequent statistical analyses on alpha and beta diversity, as well as differential functional analyses. For taxonomic analyses, only 69 and 55 samples contained more than 200 and 400 reads, respectively. Of those, only 27 and 19 pairs of samples exceeded 200 and 400 reads, respectively. The list of the subsets of samples in those four scenarios can be found in Supplementary Table S2 online. Regarding gene and functional analyses, only samples containing a minimum of 127 sequence reads of microbial origin were considered. In this case, only 58 individual samples and 19 pairs of samples contained at least 127 reads.

### Ecological diversity

The diversity within and between samples was evaluated in our paired samples containing more than 200 and 400 reads.

**Figure 1:**
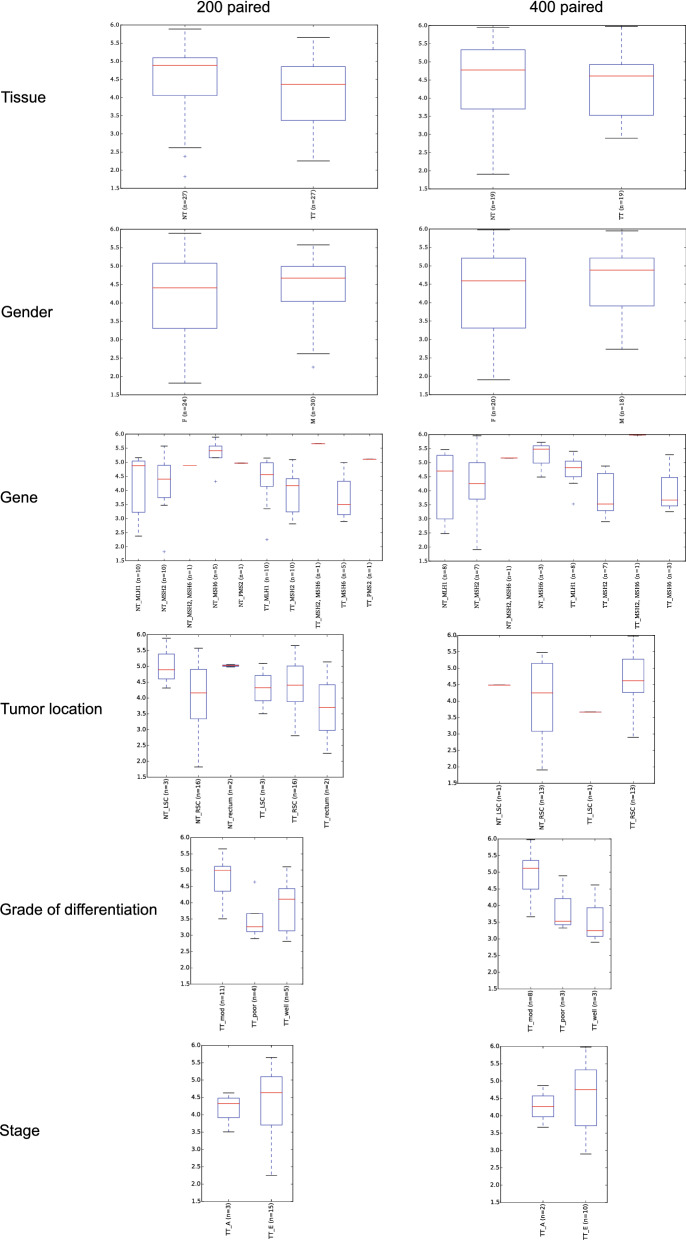
Taxonomic ecological alpha diversity analysis by tissue, gender, gene mutated, tumor tissue, tumor location, grade of differentiation and stage. Analyses are shown for paired-only samples with more than 200 and 400 reads. Diversity within samples is estimated by the Shannon diversity index. In the case of tissue and gender, the *p*-values of the pairwise comparison is shown, regardless of the significance, whereas in the case of the gene in both normal and tumor tissue, *p*-values are shown only in significant pairwise comparisons (*p*-value < 0.05). NT: normal tissue, TT: tumor tissue; F: female; M: male; LSC: left-sided colon (descending + sigmoid); RSC: right-sided colon (ascending + transverse); E: early stage (I + II); A: advanced stage (III + IV).

Our results show that the bacterial communities of our set of samples did not differ significantly (i.e. *p*-value > 0.05) in their ecological alpha diversity, estimated by the Shannon diversity index, when compared by tissue, gender, mutated gene and tumor location for each type of tissue, as well as by type of tumor and grade of differentiation (Fig. 1). Using both cut-offs of 200 and 400 reads, similar indices were obtained between normal mucosa (4.46 ± 1.02/4.43 ± 1.16) and tumor (4.16 ± 0.87/4.37 ± 0.87) tissue (*p*-values = 0.247 / 0.853), as well as between male (4.39 ± 0.86/4.58 ± 0.86) and female (4.21 ± 1.06/4.24 ± 1.13) samples (*p*-values = 0.481 / 0.315). The multiple comparisons between mutated genes (MHL1, MSH2, MSH6, PMS2) in each type of tissue only revealed occasional and meaningless differences, except for the mutation MSH6, which showed significantly lower diversity in tumor (3.78 ± 0.78) than in normal (5.27 ± 0.53) tissue (*p*-value = 0.021), but it was only significant in the case of cut-off at 200 reads. Comparisons between the different tumor locations, split according to tissue type, and grade of differentiation, did not show significant differences either, with *p*-values close to 1 in most cases. Therefore, overall, no enrichment or impoverishment of taxa was attributable to any of the evaluated variables.

**Figure 2:**
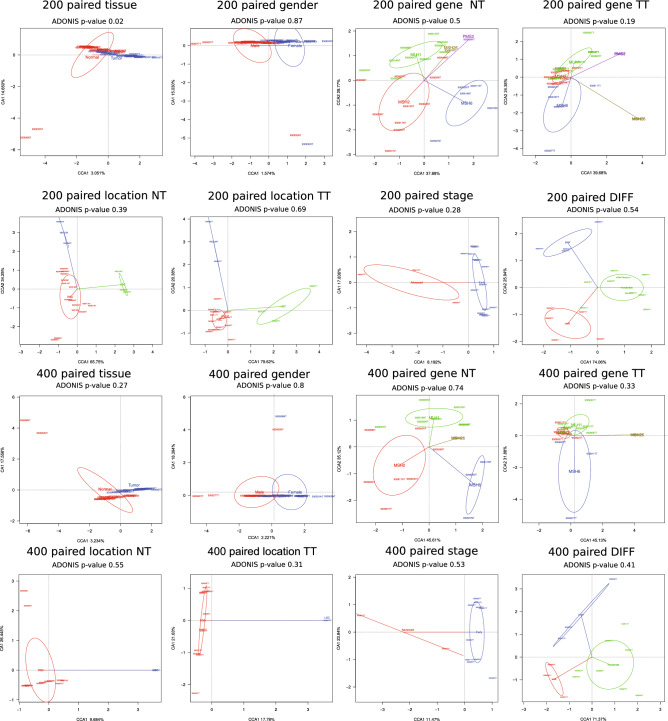
Taxonomic ecological beta diversity analysis by: tissue, gender, mutated gene, tumor location, stage and grade of differentiation. Analyses are shown for paired-only samples with more than 200 and 400 reads. Diversity among samples is shown through canonical correspondence analysis (CCA) and Adonis tests for significance. NT: normal tissue, TT: tumor tissue, DIFF: grade of differentiation.

As for the ecological beta diversity, we used the Canonical Correspondence Analysis (CCA) to elucidate the relationships between the biological assemblages of taxa and genes with environmental variables such as tissue type, patient gender, mutated gene, tumor location, the grade of differentiation, and stage, with their ADONIS *p*-values (Fig. 2). Tissue type was able to explain the distribution of taxa as separate groups between normal mucosa and tumor tissue in the set of paired samples with at least 200 reads per sample (*p*-value = 0.02), similarly to the use of all samples, without distinction of those paired and unpaired and a minimum number of reads (see Supplementary Fig. S1 online). However, with a stricter cut-off of 400 reads, this difference was not significant (*p*-value = 0.27), maybe due to the lower resolution of this smaller subset of samples. None of the remaining variables was able to explain the observed sample assembly, as no significant relationships could be established in the remaining cases in any set or subset of samples, which may be explained, at least partially, by the disproportionate number of samples per group and in many cases, the low number of samples in some groups. Despite this, the ADONIS *p*-values tended to be lower and therefore closer to significance in tumor tissue samples than in their normal tissue counterparts, possibly suggesting increased differences in the microbial community in tumors, associated with the specific gene mutation.

Overall, global differences in microbiome diversity were not significant for most of the variables considered. However, the analysis of the differential abundance between groups of samples according to those variables was able to reveal which particular taxa and genes can be associated, for example to tissue status, as we will analyze in the next section.

### Taxonomic abundance and discriminant taxa

Taxonomic characterization of the samples revealed that the relative abundance of the taxa was consistent and differences minor, regardless of the number of reads used (200 or 400), and the usage of only those samples that had both members of the pair successfully sequenced (hereafter paired) or without that restriction (hereafter paired-unpaired). Globally, Bacteroidetes (40.4%), Proteobacteria (25.9%), Actinobacteria (11.38%) and Firmicutes (11.13%) were the most abundant phyla; and *Bacteroides* (26.96%), *Burkholderia* (13.18%) and *Fusobacterium* (4.42%) the three most abundant genera. In addition, fungi from the genus *Penicillium* (phylum Ascomycota) (3.18%) were the most abundant eukaryotes described in the samples, followed by genera *Malassezia* (phylum Basidiomycota) (0.52%) and *Aspergillus* (phylum Ascomycota) (0.09%). Viral reads represented only around 0.25% of the total, with only representatives of undetermined bacteriophages from Siphoviridae family (order Caudovirales) (0.11%) and genus *Roseolovirus* (order Hespesvirales) (0.07%). Detailed information on the relative abundance of the taxa at phylum and genus level is shown in Supplementary Table S2 online.

**Figure 3:**
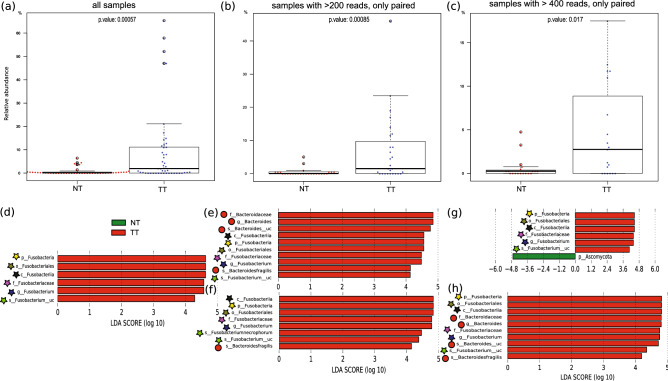
Discriminant taxa between tumors and normal mucosa. (Top) Boxplots of Wilcoxon Signed Rank tests for paired samples for *Fusobacterium* genus (top) showing statistically significant differences between normal mucosa tissue (NT) and tumor tissue (TT), for three sets of reads: all reads without normalization (**a**), paired-only samples with more than 200 (**b**) and 400 (**c**) reads. (Bottom) Linear Discriminant Analysis (LDA) Effect Size (LEfSe) plot of taxonomic biomarkers identified in the FFPE tissue microbiome from five sets of reads: all reads without normalization (**d**), paired-only samples with more than 200 reads (**e**), paired plus unpaired samples with more than 200 reads (**f**), paired-only samples with more than 400 reads (**g**), and paired plus unpaired samples with more than 400 reads (**h**). Stars indicate *Fusobacterium* lineage, circles indicate *Bacteroides* lineage.

Comparison between normal and tumor tissue revealed some coincidences but also interesting differences in the most abundant taxa. At phylum level, coincidences included the same order of the most abundant bacteria, namely Bacteroidetes (36.3% in NT and 44.59% in TT) and Proteobacteria (30.9% in NT and 20.87% in TT) and either Firmicutes or Actinobacteria in the third or fourth position. However, a deeper analysis revealed noticeable differences. For example, in NT fungi from phylum Ascomycota were approximately 56 times more abundant compared to TT (6.71% vs. 0.12% respectively), whereas phylum Fusobacteria and viruses were approximately ten and seven times more abundant in TT than in NT (10.22% vs. 1.03%; and 0.43% vs. 0.06% respectively). At genus level, *Bacteroides* (phylum Bacteroidetes, 21.48% in NT and 32.5% in TT) and *Burkholderia* (phylum Proteobacteria, 16.01% in NT and 10.32% in TT) were the two genera accounting for more reads in both types of tissue. However, the third most abundant genus in NT, the fungus *Penicillium* (6.29%) could be explained by its high abundance (> 75%) in two samples, while being virtually absent from the other samples. Its presence could therefore be attributed to contamination during storage or manipulation at the hospital of origin. Actually, it had no correspondence in TT (position 130 in abundance, at 0.05%). Similarly, the third most abundant genus in TT, *Fusobacterium* (8.34%) was located at position 27 in the NT (0.55%). On the contrary, the difference in *Fusobacterium* in favor of the TT samples corresponded to its more homogenously abundant presence in this group. In fact, statistical analyses (Wilcoxon Signed Rank tests and LefSe) support the significance of this difference by tissue type, despite using 200 or 400 reads or only paired or paired -unpaired samples (see Fig. 3) and it was the only genus showing significance in all tests. However, other taxa proved significant in some, though not all, of the tests and/or comparisons, outstandingly from the family Bacteroidaceae, such as *Bacteroides fragilis*.

**Figure 4:**
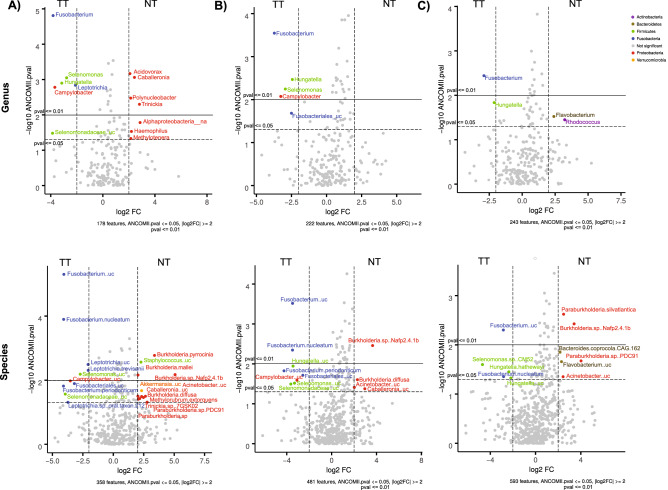
Volcano plots showing the differential abundance of taxa, at genus (top) and species (bottom)level, identified using ANCOMII analysis, between normal mucosa and tumor tissue. Three subsets of samples were used for this analysis: (**A**) all samples, (**B**) only samples with more than 200 reads in both samples in the pair, and (**C**) only samples with more than 400 reads in both samples in the pair. Features are sorted by *p* value. Significant dots showing features above *p*-value < 0.05 (dashed line) and log fold change (log2FC) >  = 2, | *p*-value < 0.01 (solid line) | log2FC ≥ 2, are colored according to the phylum they belong to.

In addition, those features differing in abundance, at both genus and species level, were determined between the tumor and normal tissue samples, using all paired samples, only paired samples with at least 200 reads in both samples of the pair, and only samples with at least 400 reads in both samples of the pair. Using a log2 Fold Change value (log2FC) ≥ 2, and two cut-off values for the *p*-value (*p*-value ≤ 0.01 and 0.05), we again observed some genera and species showing the most significant differences in abundance, in concordance with the other previously described tests (Fig. 4, and for detailed information, Supplementary Table S3 online). In particular, the most differential features included species from the genus *Fusobacterium* (phylum Fusobacteria), some of which were not unequivocally classified (*Fusobacterium* uc) but other reads classified at the species level such as *F. nucleatum* and *F. peirodonticum*, all of which were more abundant in tumor tissue. Other genera found to be more abundant in tumor tissue included *Hungatella* and *Selenomonas* (phylum Firmicutes), *Campylobacter* (phylum Proteobacteria), while *Leptotrichia* (phylum Fusobacteria) was found in all TT samples. The closest species could be determined for some genera only, such as *Hungatella hathewayi*, *Leptotrichia*.*trevisanii*, or *L. sp. oral.taxon* 212. Other genera and species, found more abundantly in normal mucosa comprised mostly members of the phylum Proteobacteria. In fact, in all samples the seven significant taxa (*p*-value < 0.05, namely, *Acidovorax*, *Caballeronia*, *Polynucleobacter*, *Trinickia*, *Haemophilus*, *Methylotenera* and unclassified Alphaproteobacteria) belonged exclusively to this phylum. However, few or no bacterial genera were identified in the case of paired samples accounting for more than 200 or 400 reads and, in the event, they belonged to other phyla, such as *Flavobacterium* (phylum Bacteroidetes) and *Rhodococcus* (phylum Actinobacteria), both in the comparisons involving more than 400 reads. However, at the species level, members of the phylum Proteobacteria accounted for most of the differential taxa in normal mucosa, notably several *Burkholderia* and *Paraburkholderia* species (e.g. *B. pyrrocinia*, *B. mallei*, *B. sp*. Nafp2.4.1b, *B. diffusa*, *P. silvatlantica*, *P. sp*. PDC91), as well as some other classified or unclassified species from the same phylum, such as *Methylorubrum extorquens*, *Trinickia.sp*. 7GSK02, *Caballeronia*_uc, *Acinetobacter*_uc. *Akkermansia*_uc, from the phylum Verrucomicrobia were also moderately abundant in all samples, whereas two *Bacteroidetes*, *Flavobacterium*_uc and *Bacteroides copr*ocola CAG.162 were in the subset of pairs of samples containing more than 400 reads.

### Functional abundance and discriminant genes

Regarding functional profiles of the microbiota, based solely on metagenomic gene repertoire, only 60 samples contained reads that could be functionally assigned. However, only in 19 pairs (38 samples) both types of tissue accounted for reads for comparison. The number of functionally annotated reads in those pairs totaled 116,175, ranging from 127 to 21,880, with an average of 3057.24 ± 4673.35 reads and a median of 1160 reads. Functional annotation at subcategory, pathway and KEGG annotation level was compared for paired samples without normalization and also for rarefied samples at 127 reads. Since a KEGG annotation can fall into more than one pathway and subcategory, combinations of more-than-one subcategory and pathways substantially increases the number of potential items. Thus, 5605 KEGG annotations fell into 1081 pathways or their combination, and, in turn into 422 subcategories or their combination in pairs without normalization, and 2142, 645 and 289, respectively in 127 subsampled reads. Regardless of normalization, the most abundant subcategories in the samples were two ‘Protein families’, ‘signaling and cellular processes’, and ‘genetic information processing’, with ~ 9–11% of all annotated functions each followed by ‘Carbohydrate metabolism’, ‘Unclassified metabolism’, ‘Amino acid metabolism’ and ‘Membrane transport|protein families: signaling and cellular processes’ ~ 4–5%. Details on the relative abundance of the functions annotated identified globally in all samples, tissue, gender, tissue mutation, tumor location, type of tumor and grade of differentiation using the paired, as well as paired plus unpaired samples, with the same number of reads per sample (n = 127), are shown in Supplementary Table S4 online.

As for functional diversity, in addition to the CCA analysis for the taxonomically annotated microbiota (see Sect. 2.2), we also carried out this analysis for gene functions (see Supplementary Fig. S2 online). As for taxonomy, tissue type was able to explain the observed groups, but only when all samples, paired and unpaired, were considered (*p*-value = 0.017), but in this case also by the grade of differentiation for all samples (*p*-value = 0.038), whereas results by other variables were not significant.

**Figure 5:**
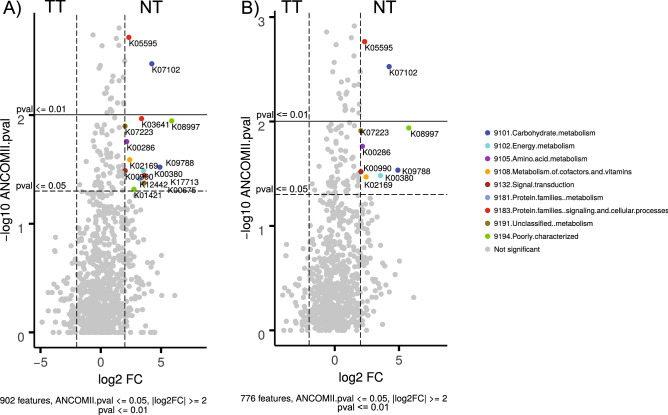
Volcano plots showing the differential abundance of gene functions identified, using ANCOMII analysis, between normal mucosa and tumor tissue. Two subsets of samples were used for this analysis: (**A**) all samples, (**B**) only samples with more than 127 reads in both samples in the pair. Features are sorted by p value. Significant dots showing features above *p*-value < 0.05 (dashed line) |log fold change (log2FC) >  = 2, and *p*-value < 0.01 (solid line) | log2FC ≥ 2, are colored according to the subcategory they belong to.

When compared by tissue type via ANCOMII, those features that differed in abundance were determined between the tumor and normal mucosa samples, using two sets of samples: (1) all samples containing at least 127 reads (58 samples) and (2) only paired samples containing at least 127 reads (38 samples). Using a log2 Fold Change value (log2FC) ≥ 2, and two cut-off values for the *p*-value (*p*-value ≤ 0.01 and 0.05), we observed 2 and 14/9 features, respectively (see Fig. 5, and for detailed information, Supplementary Table S5 online). According to this analysis, all overrepresented functions were identified to be in the normal mucosa, with no significantly overrepresented bacterial functions found in tumor tissue samples. The two most significant ones (*p*-value < 0.01) were K05595 (*marC* multiple antibiotic resistance protein) and K07102 (*amgK* N acetylmuramate 1 kinase EC.2.7.1.221) in both cases, belonging to ‘Protein families signaling and cellular processes’ and ‘Carbohydrate metabolism’ functional categories, respectively. In a second group (*p*-value < 0.05) 12 and 7 additional functions were differential between pairs of tissue samples, some of which were shared: K07223 (*yfeX* porphyrinogen peroxidase EC.1.11.1), K08997 (*ydiU* uncharacterized protein), K00286 (*proC* pyrroline 5 carboxylate reductase EC.1.5.1.2), K09788 (*prpF* 2 methylaconitate isomerase EC.5.3.3), K00990 (*glnD* protein PII uridylyltransferase EC.2.7.7.59), K00380 (*cysJ* sulphite reductase NADPH flavoprotein alpha component EC.1.8.1.2), and K02169 (*bioC* malonyl CoA O methyltransferase EC.2.1.1.197). Actually, all functions identified in the 38 paired samples were also significant in the set of 58 samples. Globally, those functions belonged to a number of different functional subcategories, with no clear dominance of a particular category.

## Discussion

Gut bacteria have been linked to CRC development, but their precise role remains unclear. This relationship has also been proposed for the specific case of Lynch syndrome^16^. A better and deeper understanding of their function when associated to the intestinal mucosa would lead to preventive medical procedures and improved cancer treatments in the future.

Studies aiming to analyze associations between gut microorganisms and mucosa have generally been carried out using fresh tissue samples. In this study, however, we used pairs of FFPE tissue samples of colorectal tumors and adjacent mucosa and applied a next-generation sequencing (NGS) metagenomic approach. The feasibility of FFPE samples for NGS analysis has been questioned because the formalin fixation process can cause disruptions in the nucleic acids such as fragmentation and mutations^17^. For this reason, we used a commercial kit (GeneRead DNA FFPE Kit, Qiagen) designed for NGS applications, that enzymatically removes artificial C > T mutations due to cytosine deamination, which are artifacts caused by formalin fixation and aging. In addition, previous studies showed that quality and feasibility of FFPE samples for NGS analysis degrade over time^17^, establishing a time lapse threshold between sample retrieval and analysis at between three and seven years. Our samples, however, encompass a wide time range, with collection dates ranging from less than one year to over 22 years’ pre-analysis.

Despite of FFPE tissue being routinely used and widely available as clinical material, its use for targeting the microbiota has been very limited due to the aforementioned drawbacks. A recent study^18^ compared the FFPE and frozen gastric tissue samples to conclude that despite similar diversity, the former showed lower number of operational taxonomic units (OTUs) and differences in relative abundance of the different phyla. However, this work was based on targeting the bacterial 16S rRNA gene, which can be affected not only by bias introduced during the embedding process and the time of tissue archiving but also in the amplification, also being limited to the evaluation of bacterial abundance profiles. Our approach, based on the shotgun metagenomic analysis of the microbial community of colorectal tissue allowed us to simultaneously respond to two questions regarding the microbial community inhabiting the FFPE tissue samples. Indeed, not only could we decipher which bacteria, fungi and viruses inhabit those samples from a taxonomic point of view, but also unravel the gene repertoire associated to those, with the advantage of minimizing the amount of DNA required, which is limited and poor in quality.

This study reports the feasibility of the analysis of the microbiota associated to tumors and adjacent biopsies from non-fresh stored FFPE samples in a particular subtype of colorectal cancer, characterized by the presence of mutations in the DNA mismatch repair system, typical of Lynch syndrome patients. Our results corroborate some findings previously reported from fresh tissue samples, namely the identification of differentially more abundant bacteria in tumor than in normal mucosa, such as *Fusobacterium* genus and *B. fragilis*^5,10^, among others.

Bacteria belonging to the genus *Fusobacterium* appeared to be most predominant and ubiquitously distributed among the tumor samples in contrast to normal mucosa. *Fusobacterium nucleatum* has been widely proposed as a causative factor of tumorigenesis^19^ and metastases^20^, although more extensive research is required. It appeared in our samples together with other genera, such as *Bacteroides*, *Selenomonas* and *Prevotella*, which are typically found to be associated with *F. nucleatum*^20^. It is worth noticing that some of these bacterial taxa found as predominant in our tumor samples, such as the species *F. nucleatum*, and species from the genera *Fusobacterium* and *Leptotrichia,* all belonging to the phylum Fusobacteria, as well as the genus *Campylobacter* (phylum Proteobacteria), are reported to have a significantly greater presence in malignant oral leucoplakia compared to healthy oral mucosa^21^. Many studies indicate that oral bacteria can be involved in CRC. Thomas and collaborators performed a meta-analysis of public databases (n = 969) of fecal samples, and reported that the oral species richness and total abundance in CRC patients was greater compared to controls, which may be a sign of an altered gut microbiota^14^. A proposed mechanism whereby these bacteria colonize the gut involves their continuously being swallowed in saliva, and their spreading via the blood-stream and systemic circulation from the oral cavity to other parts of the body, including joints, heart and colon^22^. Flemer and collaborators found that some oral taxa are differentially abundant in CRC patients compared to controls, and proposed using oral microbiota for CRC screening^23^. Interestingly, they also reported that taxa typically found in both the oral cavity and the colon, like *Fusobacterium*, were less abundant in individuals with CRC compared to healthy controls. The explanation for this still remains unclear.

Species classified within the genus *Fusobacterium* can be found in various mucosal sites throughout the human body, as well as in other animals. Their presence in healthy tissue suggests that they are members of the normal microbiota. Nevertheless, its ecological relation with mammalian tissues ranges from mutualistic or neutral to pathologic, as it is found in oral biofilms from both healthy individuals and pathological samples. It has been observed that *F. nucleatum* can induce an inflammatory response in the host, thus contributing to oral tumor progression in a disease model. Beyond this modulation of the immune system, it has the capacity to attach and invade epithelia^24^. There is no evidence to consider *F. nucleatum* as an infective agent according to Koch’s postulates. Nonetheless, due to new observations and understanding facilitated by novel technologies, together with the scientific questions answered by the advances made in the last century, these postulates are currently being updated and reformulated, and are currently experiencing a resurgence, considering the context of complex ecological relations in microbial communities and from a broad systems biology perspective^25^, and commensal host-microbiota relationship with health^26^.

The same criteria can be applied to other species such as *Bacteroides fragilis.* It encodes the *B. fragilis* toxin (BFT), a zinc-dependent metalloprotease which degrades E-cadherin and facilitates the nuclear translocation of β-catenin and transcription of Myc proto-oncogene, provoking cellular proliferation in the colon epithelium^27^. LEfSe analysis in our study showed higher abundance of *B. fragilis* in tumors than in normal mucosa. Dejea and collaborators reported a greater presence of *B. fragilis* and *E. coli* in colonic biofilms in patients with familial adenomatous polyposis (FAP), a hereditary condition which, like Lynch syndrome, increases the risk of developing CRC^28^. In that case, *B. fragilis* was found in the mucosa of patients with precancerous benign lesions (polyps) but not CRC, being the control group non-FAP healthy individuals. Expression of bacterial oncotoxins-coding genes like *bft* were also augmented in FAP patients compared to controls, and the experiments with APC mutant mice demonstrated that this taxon plays a role in CRC development. In contrast to this, Lee and collaborators found that *B. fragilis* shows a protective effect in a mouse model for colitis-associated CRC induced with azoxymethane (AOM)/DSS, and suggested the use of *B. fragilis* as a preventive therapeutic strategy against inflammatory CRC^29^.

Belcheva and collaborators demonstrated the carcinogenic potential of gut bacteria in APC^Min/+^MSH2^−/−^ mice, a model for HNPCC or Lynch syndrome, which was independent of the inflammatory process, and based on carbohydrate metabolism, from bacterial degradation of fiber and butyrate production^15^. They showed that butyrate, a short-chain fatty acid (SCFA), promotes epithelial cell proliferation and transformation in mice with this genetic profile. The causative mechanism of carcinogenesis in this case is based on the alteration of proliferative responses. We identified bacteria in the tumor tissue that promote intestinal inflammation, such as *F. nucleatum* and *Campylobacter*. Both of the abovementioned mechanisms, inflammation and alteration of cell proliferation, are not mutually exclusive. Butyrate production by some bacteria might promote carcinogenesis through aberrant proliferation and transformation. After this, or at the same time, some other bacteria that are opportunistic pathogens, such as *F. nucleatum* and *Campylobacter*, might enhance the process in the affected locations through alteration of signaling routes and inflammation.

Both inflammation and immunodeficiency increase the probability of developing cancer. LS patients have a higher risk of suffering cancer due to their mutations in MMR genes and the consequent accumulation of secondary mutations. Therefore, it would be reasonable to expect that immunodeficiency, added to LS condition, may have a great negative impact in these patients. Zheng and collaborators^30^ studied the effect of immunodeficiency in the gut microbiome in mice models, and found that SCFA-producing and fiber-degrading bacteria were more abundant in immunodeficient mice. This was interpreted as a microbial compensation and alleviating for immune barrier deficiency. These taxa are considered as beneficial for gut homeostasis but, as mentioned above, it can be a different case for LS individuals. It would be interesting to study in the future the effect that the immune system may have in the gut microbiota of MMR model mice.

*Campylobacter* is another very relevant genus we found to be significantly associated with tumors and widely present among our study cohort. It was also found in feces of CRC patients as differentially abundant compared to controls^14^. Due to the short length of the DNA fragments due to DNA degradation caused by the effect of formalin fixation and paraffin embedding, we were unable to identify at the species level with enough confidence. Luethy and collaborators proposed that *C. jejuni* uses the spatial gradient of microbiota-generated SCFAs as a cue to select the preferred niches in the lower intestinal tract and SCFAs also induce the gene expression necessary for mucosal colonization^31^. Although they studied this process in avian hosts, they speculated that the mechanism could be similar in humans. *Campylobacter spp*. has been associated to CRC development. In particular, *C. jejuni,* as well as other pathogenic bacteria, produces cytolethal distending toxin (CDT), a genotoxin with DNA damage potential that can promote intestinal inflammation and colorectal cancer^32^. In fact, the presence of CDT-producing *C. jejuni* appears to affect the gut microbiota composition and its functional expression^32^. *Campylobacter* and *Fusobacterium* together were reported as the key genera of tumor microbiota in a small cohort of 19 patients harboring colorectal cancer^33^.

The abundance of the genus *Hungatella* was also significantly higher in tumor samples compared to normal mucosa. Thomas and collaborators found a variant of the *cutC* gene belonging to *Hungatella hathewayi* and *Clostridium asparagiforme* (phylum Firmicutes), which was strongly associated to CRC, while other variants, synthesized by other taxa, were not^14^. This gene is implicated in the synthesis route of trimethylamine (TMA), an amine which plays a role in other pathologies as well, and is a gut-bacteria product derived from choline. They proposed that *cutC* variants mostly belonging to *H. hathewayi* and *C. asparagiforme* were significantly associated to CRC and other diseases, probably due to differences in the efficacy of choline degradation and TMA production.

The aforementioned work and another similar study performed by Wirbel and collaborators^15^ found some taxa in common with those present in our study, like *F. nucleatum* and *H. hathewayi.* Although Flemer and collaborators found that fecal microbiota reflected only partially the mucosal associated microbiota in CRC patients, it is interesting to consider these results, as disease-related differences in the microbiota are evident in fecal samples^13^.

Another relevant agent able to induce mutations in epithelial cells DNA is a bacterial genotoxin called colibactin, produced by certain strains of *Escherichia coli* and other members of the family Enterobacteriaceae. The genes encoding it are found in the *pks* genomic island. Colibactin-producing bacteria have been related to CRC development in human and mouse models. Recently, a study^34^ demonstrated a link between colibactin exposure of human intestinal epithelial cells and two mutational fingerprints found in colorectal tumors. These fingerprints characteristics are consistent with DNA cross-links, adducts and alkylation described in previous research^35,36^. Pleguezuelos-Manzano and collaborators^34^ developed human intestinal organoids and exposed them to genotoxic *pks* + *E. coli*. Finally, they performed whole-genome sequencing of the organoids and found these mutational signatures, also present in colorectal tumors from two independent large cohorts of patients, but not in other types of cancers, as an evidence of the role of bacteria in colorectal carcinogenesis.

Interestingly, we were able to identify some reads in several samples mapping against the *pks* genomic island from *E. coli* strain IHE3034 in our metagenome samples by blast search. In particular, with the genes *clbF*, *clbO*, *clbC* and *clbI*, and other intergenic sequences, with high identities, between 98 and 100%. We also detected reads matching some of the aforementioned bacterial toxins, such as *Bacteroides fragilis* toxin (*bft*), and the variant of the *cutC* gene belonging to *H. hathewayi* and *Enterocloster asparagiformis (a.k.a. C. asparagiforme*), with identities ranging from 91.8 to 100%.

We observed other differences in bacterial composition between tumor and normal mucosa samples in our results. For example, *Akkermansia muciniphila*, which belongs to phylum Verrucomicrobia, was differentially more abundant in the normal mucosa. *A. muciniphila* has been described as a potentially beneficial microorganism in the normal microbiota and is also being considered for probiotic administration in the future^37,38^. Although it has some pathogen-like characteristics, like adhesion to mucus and mucus degradation, it does not infect the epithelium as it never reaches the inner layer of the mucosa, remaining on the outer layer only superficially^37^. A low abundance of *A. muciniphila* has been shown to correlate with diseases such as type-2 diabetes, obesity and inflammatory bowel disease. Despite its beneficial potential, it is not an unexpected finding in colorectal cancer patients. It was reported that *A. muciniphila* was four-fold higher in a cohort of colorectal cancer patients in comparison to a heathy cohort^39^. The explanations for this could rely on two conditions of colorectal cancer patients: lower food intake, characteristic of these patients’ dietary behavior, is correlated with an increase in *A. muciniphila*, and cell proliferation promotes mucus production and this could also augment *A. muciniphila*^37^.

Some other taxa that are more abundant in healthy mucosa have been described as associated to different pathologies or as opportunistic pathogens and might be ecological precursors of other bacterial taxa that we found associated to tumors. This would hint at a generalized dysbiosis in the gut microbiota associated to the mucosa of these patients in the normal mucosa, not only in situ in the microbiota of tumors. This is the case, for instance, of *Burkholderia*, a genus which includes species that can produce a variety of infections, including pulmonary conditions. They are opportunistic pathogens that can produce biofilms on epithelial tissue, thus being harder to eradicate^40,41^.

Regarding the functional profile little differentiation between healthy and tumor tissues was apparent, maybe partly due to the low number of samples. One possible explanation to the observed absence of significant differences in differential gene function among tumors might be due to the singular natural history of tumors in the Lynch syndrome context. The molecular hallmark of virtually all Lynch syndrome tumors is the presence of microsatellite instability (MSI) due to inactivating biallelic mutations on DNA mismatch repair genes. MSI defines a specific molecular pathway in colorectal carcinogenesis with a very homogeneous tumor behavior at different levels: (1) molecular, with the presence of thousands of mutations in tumor cells (hyper-mutated tumors); (2) pathological, tumors are very immunogenic, with typical strong lymphocyte infiltration in tumors, that might impact on the microenvironment, and consequently, on the microbiota; (3) clinical, tumors with better prognosis and with a good response to immunotherapy. In this scenario, the apparent homogeneity in differential gene function is not surprising, although other possible explanations may also come into play. Despite this, it is worth noticing the significantly reduced carbohydrate metabolism in tumor tissue samples. It is related to the expression of the gene *amgK* that encodes for the N-acetylmuramate/N-acetylglucosamine kinase. It is a sugar kinase that catalyzes the ATP-dependent phosphorylation of N-acetylmuramate (MurNAc) and N-acetylglucosamine (GlcNAc). It is involved in anabolism and biosynthesis, specifically in the pathway for peptidoglycan recycling, which is part of cell wall biogenesis.

One inherent weakness of this study is that, after applying bioinformatic quality control, the number of reads was considerably low for some samples, especially after the removal of reads of human origin. Although the number of samples collected for our study would be considered acceptable in the case of fresh feces or even fresh tissue, maybe it should be higher in the case of FFPE samples in order to achieve better statistical power, since the low quality of the DNA of this sample type means that a significant number of them are rejected due to the dearth of microbial reads retrieved. There may be different reasons for this, such as aging due to the time elapsed since sample retrieval or formalin fixing time, which might have been insufficient. Thus, we recommend the use of larger sample sizes when utilizing FFPE samples, in order to overcome these possible drawbacks. Nevertheless, our findings are consistent with previous studies, as mentioned above. Another possibility that can, at least partially, explain the low number of microbial reads in some samples is that in this study we were unable to choose the location of the cuts in the paraffin-embedded tissue blocks, which were sent from different hospitals. It is normally not possible to distinguish, orientate and select the most apical side in paraffin blocks in the case of colorectal tumors for cutting the slides of tissue.

Overall, alpha and beta diversities do not differ significantly by tissue, gender nor mutated gene. However, this must be taken with caution because, although the general trend is an evenness of taxa for these variables, the limited number of bacterial reads appears to contribute to the apparent lack of differences between them for both types of analyses. The results were not significant for the statistical analysis comparing the right and the left sides of the colon. One explanation for this lack of significance can be the disparity in size between subgroups, with some of them consisting of very few samples compared to others. This was, in turn, due to the limitations imposed by samples that were collected from existing collections, which prevented us from a design involving a selection ad hoc of samples that were balanced in terms of tumor location, mutation, type of tumor, gender, and other variables. It would be recommendable, if possible, to select cases for setting up subgroups of comparable sizes.

In the future, it would be interesting to compare tissue samples from colorectal cancer patients diagnosed with Lynch syndrome and samples from healthy volunteers who donate a small sample during control colonoscopy, as well as with patients with other types of CRC.

It could also be worth checking whether it is possible to find taxa that are differentially associated to tumors in feces of high-risk cancer patients from a healthy Lynch syndrome cohort. For this purpose, it may be necessary to use quantitative PCR. This could be useful towards the development of preventive strategies.

Furthermore, the analysis of the oral microbiota of high-risk cancer patients, like LS individuals, could provide possible clues to infer the risk of an individual for developing CRC.

In this study, we corroborate the presence of taxa differentially associated to tumors in comparison to adjacent mucosa previously reported in other articles, and the feasibility of FFPE tissue samples for performing this kind of study. We also recommend increasing the sample size and the sequencing depth of research projects using FFPE samples compared to the number necessary when utilizing fresh tissue. We encourage the metagenomic analysis comparison between paired FFPE and frozen tissue samples to assess the validity of the results from FFPE samples, since they may be not optimal as a first choice, but complementary and, in cases when fresh samples are unavailable, they may represent an alternative for retrospective microbiome analysis on colorectal cancer and other pathologies.

## Materials and methods

### Patients recruitment and sample collection

Samples and data from patients included in this study were provided by the Biobank IBSP-CV (PT17/0015/0017), integrated in the Spanish National Biobanks Network and in the Valencian Biobanking Network.

The four inclusion criteria for patient selection were the following: (1) Patients diagnosed and surgically treated for colorectal cancer; (2) genetic diagnosis of Lynch syndrome (germline mutation in any of the genes responsible: MLH1, MSH2, MSH6, and PMS2; (3) informed signed consent for Biobank; and (4) availability of normal and tumor tissues.

Patients of the Program of Genetic Counseling in Cancer of the Valencian Community (Spain), who fulfilled inclusion criteria 1 and 2 were selected by the Valencian Network of Biobanks from the listed cases that also met the inclusion criteria 3 and 4, the diagnosis of Lynch syndrome genetics of all these patients was the responsibility of the Molecular Genetics Unit of the General University Hospital of Elche. The study design included a number of around 50 patients, taking two samples per patient, therefore 50 normal colon mucosa and 50 colon carcinomas.

Samples consisted of paired tumor tissue and normal colorectal mucosa, taken from a distance of at least 10 cm from the tumor, fixed in formalin and embedded in paraffin. Samples came from the tissue area on the cutting surface of approximately 1 cm. A total of five serial cuts of 10 microns per sample were placed in sterile screw-cap microtubes and stored at room temperature until use.

### DNA extraction

The samples were dispatched to the FISABIO facilities for processing, through the GeneRead DNA FFPE Kit (Qiagen Inc., USA), which allows the purification of genomic DNA from tissue samples fixed in formalin and embedded in paraffin, for efficient high-throughput sequencing, since it reverses crosslinks in the DNA (deaminated cytosine residues) using the enzyme uracil-N-glycosylase contained in the kit. DNA was extracted from FFPE samples using the kit, according to the manufacturer’s instructions.

### Library preparation and sequencing

Libraries were prepared using NEBNext Ultra II FS DNA Library Prep Kit for Illumina’ kit (New England Biolabs) following the manufacturer’s protocols with some additional modifications. This kit converts a wide range of DNA sizes into high quality libraries, which is convenient for these types of samples that have fragmented DNA due to paraffin. Modifications included a shorter time, two minutes, for DNA fragmentation since these samples were already highly fragmented. dAMP (dA) was added to the blunt end of the DNA fragment by PCR, thus allowing subsequent ligation of adapters. To add these adapters a PCR was performed and therefore the adapter with the complementary dT was attached. Through an incubation in the thermocycler with the enzyme USER (Uracil-Specific Excision Reagent), its endonuclease action allowed the excision of the uracil base, and consequently of the two DNA sequences. The ligation reagents were then removed with a clean-up with NucleoMag NGS Clean-up and Size Select Beads that binds to the DNA (150–800 bp). To add the ‘Barcode’ and the P5 and P7 sequences at the ends, another amplification step was carried out. The libraries generated were quantified using Qubit dsDNA HS Assay Kit (TermoFisher Scientific). Equimolar concentrations of the sample libraries were pooled and sequenced in NextSeq500 sequencer (Illumina) with 150 bp paired-end chemistry, at the facilities of the Sequencing and Bioinformatic Service of FISABIO (Valencia, Spain).

### Data processing

The bioinformatic analysis of the resulting sequence reads included a first step of artifact filtering and sequence trimming of low average quality (Q < 30), and sequences shorter than 50 bp, using PRINSEQ-lite (v0.20.4)^42^. Adapter sequences at the 3′-end of the reads were removed with Cutadapt v2.0^43^. Then the direct and reverse sequences were joined with the FLASH program^44^, applying the default parameters. Sequences of human origin were removed with the bowtie2-2.3.4.1 program^45^, mapping against the reference database of the human genome (GRCh38.p11, reference human genome, Dec 2013) with local and very sensitive options.

Functional annotation analyses were carried out on the human-free sequences obtained after bowtie2 filtering. First, reads were assembled for each sample by MEGAHIT v1.1.2^46^, and reads were aligned to the resulting contigs to identify which reads assemble. Those that did not assemble were appended to the contigs for the gene prediction program Prodigal v2.6.3^47^. Functional annotation was carried out with HMMER^48^ against the Kyoto Encyclopedia of Genes and Genomes (KEGG) database version 2016^49^ to obtain the functional subcategory, pathway and annotation of the genes. The filtering of the best annotations and the assignment of the open reading frames (ORF) to every read were carried out using the statistical package R 3.1.0^50^, which also was used for counting the aligned reads and for addition of the category and its coverage, and finally to create contingency tables of abundance.

In parallel, taxonomic annotation of the sequences was carried out by assigning each reading of each sample using Kaiju v1.6.2^51^ against the non-redundant NCBI database. From there, each reading inherited the consensus taxonomy from its corresponding and best alignments following the LCA algorithm (Last Common Ancestor), with the statistical package R, to generate the taxon abundance table.

Finally, the QIIME pipeline version 1.9.0^52^ was used in the statistical analysis, which allows the microbiome to be analyzed from raw sequencing data and generates quality graphs (bar charts). For the alpha diversity or diversity within the samples, the Shannon diversity index, an index that measures how informative the composition of a sample is, was calculated using the script alpha_diversity.py. Statistical differences in that index between pairs of groups of samples were analyzed using the script compare_alpha_diveristy.py, which also generated the corresponding boxplots. As for beta diversity, variation was assessed using the multivariate method canonical correspondence analysis (CCA) including the calculation of their corresponding Permutational Multivariate Analysis of Variance or Adonis values for groups, conducted on a Bray–Curtis dissimilarity matrix created using R version 3.6^50^. To identify taxonomic biomarkers between the normal and tumor tissue, we applied the linear discriminant analysis effect size (LEfSe) method, combining the Kruskal–Wallis and pairwise Wilcoxon tests for statistical significance with linear discriminate analysis for feature selection, to confirm the differential abundance taxa. We used default significance (alpha value = 0.05) and linear discriminant analysis threshold (4.0), at all taxonomic levels^53^. Differential abundance in genera/species and KEGG annotated genes across samples by tissue and gender were determined using ANCOM and Wilcoxon-Mann–Whitney non-parametrical tests, also conducted using R package, and volcano plots were plotted using the EnhancedVolcano package of R.

### Ethics approval and consent to participate

All experimental protocols, as well as the sample cession were approved by the Ethics Committee for Clinical Research of the Directorate General of Public Health and Center for Advanced Research (CEIC-DSP / CSISP). The study was conducted in accordance with the Declaration of Helsinki and other relevant guidelines and regulations.

As samples were taken from humans, written informed consent was obtained from all patients involved in the study before participation.

## Supplementary information

Supplementary Figures.

Supplementary Tables.

## Data Availability

The metagenomes data sets from this study are available in the EBI Short Read Archive under the study accession number PRJEB34333, with accession numbers [ERS3740403- ERS3740499].
