# Supplementary material for: Metagenomic analysis of formalin-fixed paraffin-embedded tumor and normal mucosa reveals differences in the microbiome of colorectal cancer patients
Source: Sci Rep. 2021 Jan 11;11:391. doi: 10.1038/s41598-020-79874-y (PMC7801721; doi:10.1038/s41598-020-79874-y)
Supplement: Supplementary file 1 — Supplementary Figures. [file 41598_2020_79874_MOESM1_ESM.pdf]

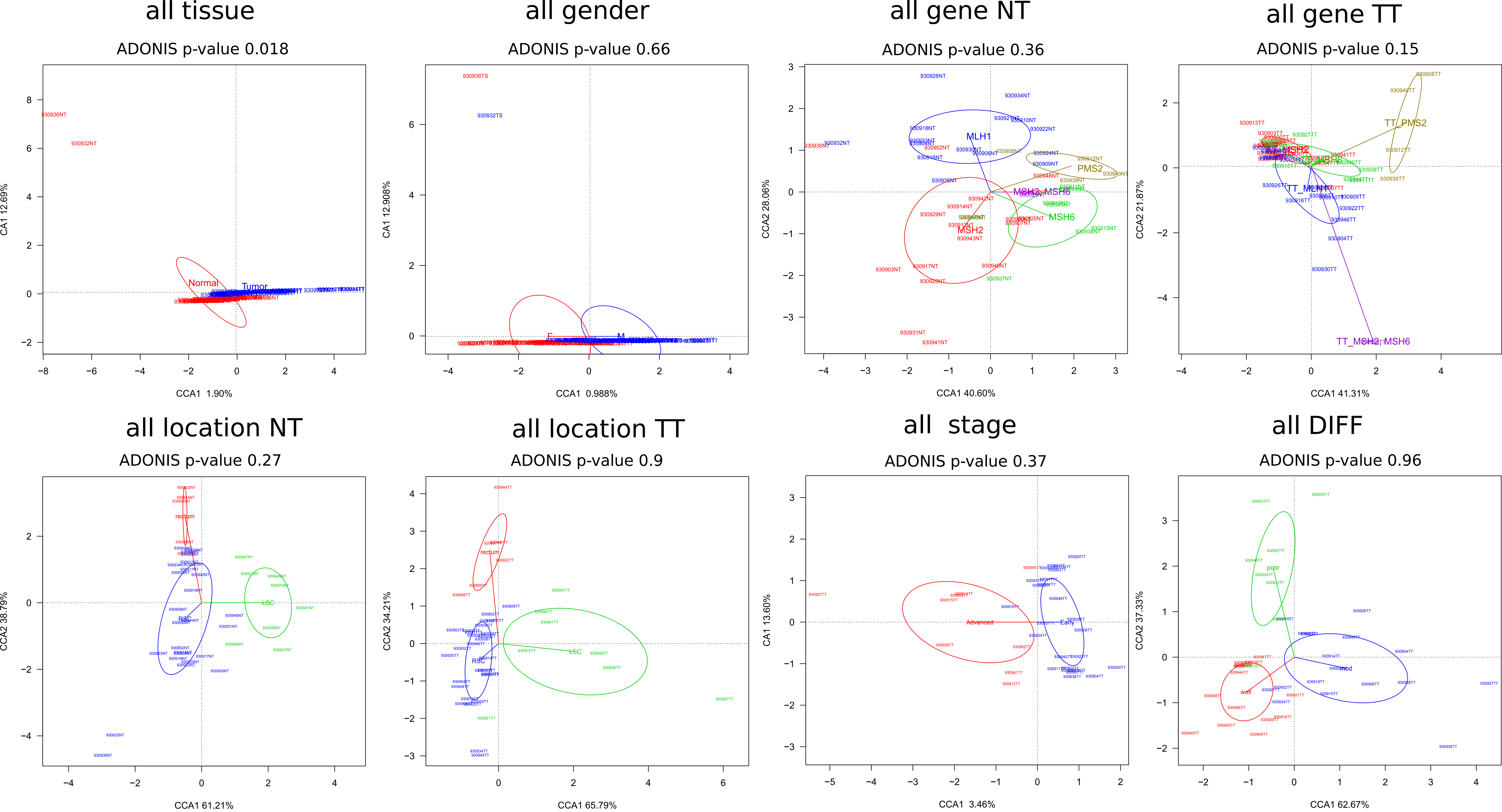

Supplementary Figure S1. Canonical correspondence analysis of all, paired, and unpaired samples, with no further filtering and normalization step, based on the taxonomy, for the tissue type (normal mucosa tissue vs. tumor tissue), gender of the subjects (females vs.males), mutated gene and tumor location, with distinction between normal and tumor tissue, and by grade of differentiation (poorly, moderately and well differentiated) and stage (early vs. advanced) for tumor tissue samples.

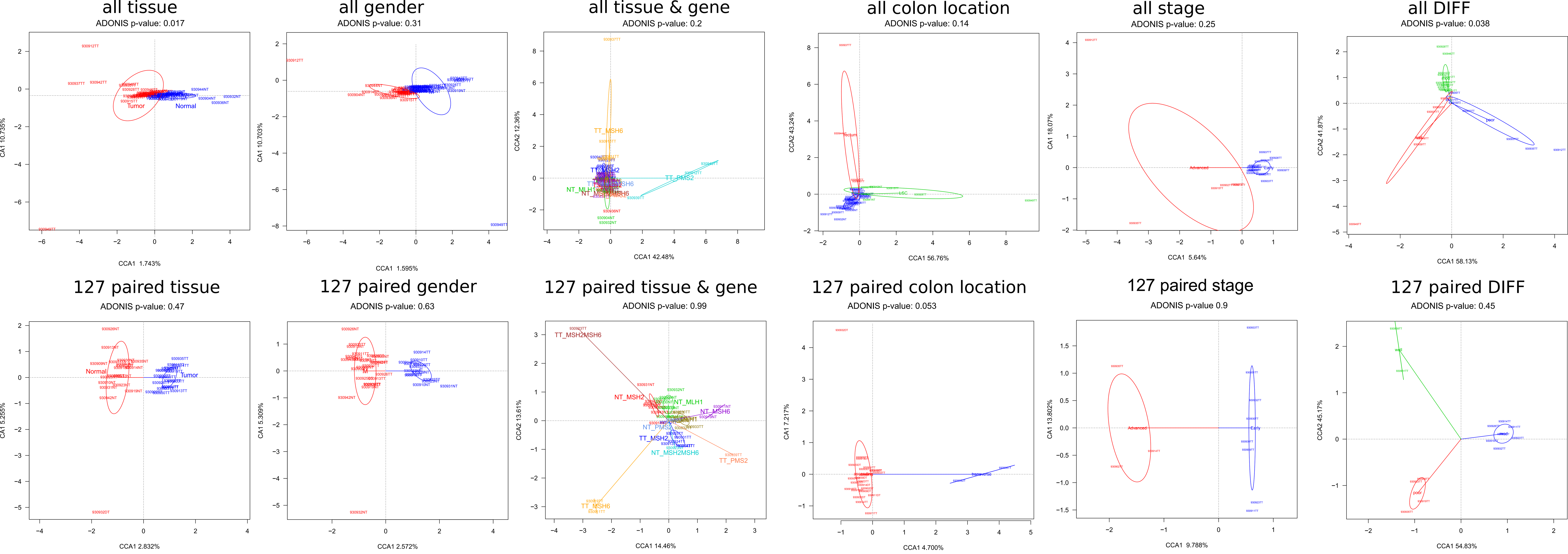

Supplementary Figure S2. Canonical correspondence analysis of all, paired and upaired samples, as well as of paired-only samples with more than 127 reads, based on function, for tissue type (normal mucosa tissue vs. tumor tissue), gender of the subjects (females vs. males), gene mutated for normal and tumor tissue, stage (early vs. advanced) and by grade of differenciation (poorly, moderately, and well differenciated) for tumor tissue samples . Statistical significance is indicated by the ADONIS tests p-values.
